# Adapting the design of the ongoing RAMPART trial in response to external evidence: An example for trials which take many years to run and report

**DOI:** 10.1016/j.conctc.2024.101381

**Published:** 2024-10-18

**Authors:** Angela Meade, Elena Frangou, Babak Choodari-Oskooei, James Larkin, Tom Powles, Grant D. Stewart, Laurence Albiges, Axel Bex, Toni K. Choueiri, Ian D. Davis, Tim Eisen, Alison Fielding, Craig Gedye, David J. Harrison, Rick Kaplan, Salena Mulhere, Paul Nathan, Grisma Patel, Jay Patel, Hannah Plant, Alastair Ritchie, Hannah Rush, Clare Shakeshaft, Martin R. Stockler, Cristina Suarez, Jemima Thompson, Nat Thorogood, Balaji Venugopal, Mahesh K.B. Parmar

**Affiliations:** a MRC Clinical Trials Unit at UCL, Institute of Clinical Trials & Methodology, 2nd Floor 90 High Holborn, London, WC1V 6LJ, UK; b Royal Marsden Hospital, Royal Marsden Hospital, 203 Fulham Rd, Chelsea, London, SW3 6JJ, UK; c St Bartholomew's Hospital, W Smithfield, London, EC1A 7B, UK; d University of Cambridge, Department of Surgery, University of Cambridge, Cambridge Biomedical Campus, Cambridge, CB2 0QQ, UK; e Institut Gustave Roussy, 114 Rue Edouard Vaillant 94805, Villejuif, France; f Royal Free London NHS Foundation Trust UCL Division of Surgery and Interventional Science, Pond Street, London, NW3 2QG, UK; g Netherlands Cancer Institute, Amsterdam, the Netherlands; h Dana-Farber Cancer Institute, 450 Brookline Ave, Boston, MA, 02215, United States; i Monash University Eastern Health Clinical School, Level 2, 5 Arnold Street, Box Hill, Victoria, 3128, Australia; j Department of Medical Oncology, Eastern Health, Melbourne, Australia; k Department of Oncology, Cambridge University Hospitals NHS Foundation Trust, Addenbrooke's Hospital, Cambridge Biomedical Campus, Hill's Road Cambridge, CB2 0QQ, UK; l Faculty of Health and Medicine, The University of Newcastle, Australia; m University of St Andrews, North Haugh, St Andrews, KY16 9TF, UK; n Mount Vernon Cancer Centre, Rickmansworth Rd, Northwood, HA6 2RN, UK; o NHMRC Clinical Trials Centre, University of Sydney, Camperdown, NSW, 2006, Australia; p ANZUP Cancer Trials Group, Sydney, Australia; q Vall d'Hebron Institute of Oncology, Vall d'Hebron University Hospital, 08035, Barcelona, Spain; r School of Cancer Sciences, Beatson Institute, University of Glasgow, G61 1BD, UK

## Abstract

Clinical trials to establish the efficacy of new agents in the adjuvant cancer setting typically take many years to complete. During that time, external factors can impact recruitment and reporting plans. An example is a new standard of care becoming available during the recruitment period.

In this paper we describe how we modified the design of the RAMPART trial (NCT03288532) which was set up to investigate immune checkpoint inhibitor therapy in the adjuvant renal cancer setting. The trial had been initiated when no globally accepted adjuvant strategy after nephrectomy existed. A subsequent change in the standard of care for many patients with early renal cancer meant it was no longer feasible to continue to recruit. We needed to find a way to maximise the contribution that RAMPART participants could make to the evidence base for immune checkpoint inhibitor therapy without introducing bias or detriment to the integrity of the trial results. We describe how we agreed and incorporated all design and timeline changes while remaining blinded to accumulating data within the trial, thus protecting the reliability of the future results. We share details of our design modifications to guide others who may have similar experiences, particularly as more agents and combinations of agents are developed and investigated in similar adjuvant settings.

## 1 Introduction

Clinical trials to establish the efficacy of adjuvant therapies for early cancers are lengthy and challenging undertakings, typically involving a large sample of patients and an extended follow-up period. They consequently take many years (typically more than 5) from start to finish.

Unanticipated external events may arise during the conduct of a trial which affect the original intentions and context. One such example is new treatment options and/or new standards of care becoming available during the recruitment period. If this situation arises well before recruitment is complete but after the trial organisers – and research participants – have invested several years of work, a dilemma exists. If the trial outcome is still important, then abandoning the trial too early may jeopardise its power to detect clinically meaningful effects. In addition, interim (early) looks at the data can increase the chance of a false positive result if not carried out in an appropriate and statistically principled way. Nevertheless, the trial organisers have an obligation to the trial participants to maximise what can be learned and concluded from the trial while retaining the integrity of any results. This is the situation we faced with the Renal Adjuvant Multiple Arm Trial (RAMPART (NCT03288532)), an international investigator-initiated phase III multi-arm multi-stage (MAMS) randomised-controlled platform trial in the post-nephrectomy adjuvant renal cell cancer (RCC) setting [1,2]. In this paper we discuss how we modified the design of RAMPART in response to new, external results and, subsequently, the change in standard of care for many patients with early renal cancer. We also describe how we have been able to do this without introducing bias, thereby retaining the integrity of future results.

## 2 Original RAMPART design

RAMPART was designed as a three-arm trial, permitting the investigation of the PD-L1 inhibitor durvalumab as monotherapy (arm B) and separately in combination with the CTLA-4 inhibitor tremelimumab (arm C) compared with a common control arm of active monitoring (arm A).

The RAMPART MAMS platform design permits the evaluation of multiple treatments simultaneously, while offering the ability to adapt to a changing landscape as data on different agents and combinations of agents emerges. At the outset, the multi-stage design included pre-planned, time-to-event driven interim analyses for both lack of benefit (LoB) and overwhelming benefit (OB). LoB analyses allow the cessation of recruitment to treatment arms where research treatments potentially offer little or no benefit, which means participant accrual can be focused on the more promising research arms and the control arm. The planned OB analysis allows the reporting of results earlier if sufficient benefit is observed. The timelines for all analyses and stopping guidelines as planned at the outset of the trial are described in detail in the trial protocol, a protocol paper and a paper describing the rationale for the trial [[1], [2], [3]]. The platform design also included the intention of adding at least one more research arm in the future. Funding and drug for patients was provided by 10.13039/100004325AstraZeneca. RAMPART was designed with two co-primary outcome measures, disease free survival (DFS) and overall Survival (OS). DFS is defined as the interval from randomisation to first evidence of local recurrence, new primary RCC, distant metastases, or death from any cause. OS is the time from randomisation to death from any cause (including RCC). The target hazard ratios (HR) for the primary DFS outcome agreed at the outset of RAMPART were 0.70 for the combination of durvalumab and tremelimumab (arm C) versus control (arm A), and 0.75 for single agent durvalumab (arm B) versus control (arm A). RAMPART commenced with the plan to randomise 1750 participants over 5.5 years. The primary DFS results for the combination research arm were expected when 276 control arm events had occurred (approx. 6.25 years after recruitment started), while the results from the durvalumab monotherapy research arm would follow when 416 control arm events had occurred approx. 4.25 years later (Table 1).

**Fig. 1:**
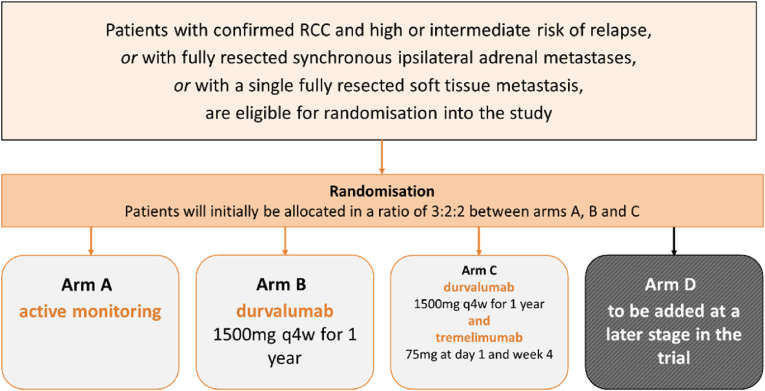
Rampart trial design.

Eligible participants were randomised in a 3:2:2 ratio between the control arm and the two research arms; 3:2:2 being considered the near optimal allocation ratio for a three-arm design [3]. Participants in the control arm (arm A) do not receive any adjuvant treatment but are actively monitored according to the same schedule as participants randomised to the research arms (Fig. 1).

## 3 RAMPART oversight

RAMPART is sponsored by 10.13039/501100000765University College London (UCL). The Medical Research Council Clinical Trials Unit (MRC CTU) at UCL has overall responsibility for the trial, working closely with the Chief Investigator and an international trial management group (TMG) of clinicians, trial co-ordinators and participant representatives. The TMG meet regularly to discuss progress and issues as they arise. An Independent Data Monitoring Committee (IDMC) meet approximately annually to review safety, compliance with treatment and efficacy data (at pre-planned interim analyses). They are the only group who see the confidential, accumulating efficacy data for the trial, although no efficacy analyses have yet to be performed. The IDMC advise the Trial Steering Committee (TSC). The TSC provides overall supervision of the trial and provides advice and recommendations through its independent Chair.

## 4 Changes to treatment options in the adjuvant RCC setting

Active monitoring was the accepted global standard-of-care after surgical resection at the outset of RAMPART and was chosen as the control arm for the trial [4]. In 2021, the KEYNOTE-564 (NCT03142334) trial was the first trial to report results, at its first planned interim analysis, for a checkpoint inhibitor as an adjuvant therapy for participants (n = 994) at high risk of disease recurrence [5]. Pembrolizumab, after surgery, resulted in a significant improvement in DFS compared with placebo; a hazard ratio for DFS of 0.63 (95 % CI 0.50–0.80) was observed with 30.1 months follow-up in results published in September 2022 [6]. Overall survival results from KEYNOTE-564 were subsequently reported; a significant improvement in OS was observed with pembrolizumab (HR for death 0.62; 95 % CI, 0.44 to 0.87; P = 0.005) [7]. This result was not known at the time of the decision to close to recruitment and therefore was not part of the decision-making process. Contrary to the KEYNOTE-564 results, data from two other large, randomised trials do not support the use of either atezolizumab monotherapy (IMmotion010) or a combination of nivolumab and ipilimumab (Checkmate 914) in the post-nephrectomy adjuvant setting. The IMotion010 trial (n = 778) compared atezolizumab with placebo and no evidence of difference in terms of DFS was observed (HR 0·93, 95 % CI 0·75-1·15, p = 0·50) [8]. Similarly, the Checkmate 914 trial (n = 816) which compared nivolumab plus ipilimumab against control showed no evidence of difference in DFS (HR 0·92, 95 % CI 0·71–1·19; p = 0·53) [9] with a median follow-up of 37 months. The 2022 European Association of Urology (EAU) guidelines, consequently, reported a weak recommendation for the use of adjuvant pembrolizumab for patients with high-risk clear cell RCC until final OS results are available [10,11].

Based on the KEYNOTE-564 results, pembrolizumab was approved by the European Medicines Agency (EMA) and US Food and Drug Administration (FDA) for the treatment of RCC in the post-nephrectomy adjuvant setting. Recommendations for reimbursement from the National Institute for Health and Care Excellence (NICE) and the Scottish Medicines Consortium (SMC) meant that, from October 2022, pembrolizumab became available in the UK as a treatment option for many of the patients who would be eligible for RAMPART.

## 5 RAMPART accrual

The first participant was randomised to RAMPART in October 2018. As for many trials, the COVID-19 pandemic had a significant impact on trial recruitment, by affecting UK site recruitment and delaying the start of international recruitment. International sites came on board from July 2021; France first, followed by Australia and Spain. Nonetheless, recruitment was consistently good throughout the first three quarters of 2022, and the TMG were hopeful that the trial would continue while it remained a valid option for potential participants. However, it became apparent that recruiting the original target of 1750 participants would take an infeasibly long period of time and after much discussion with AZ it was agreed to close to recruitment earlier than planned on June 30, 2023. This decision was confirmed in January 2023 and communicated to sites in May 2023. In total, 790 participants were randomised across the four participating countries by the time recruitment was stopped.

## 6 Modifications to the RAMPART design parameters and analysis timeline

Considering the availability of pembrolizumab and the impact of an earlier closure on the observed RAMPART sample size, modifications to the original trial design parameters were necessary to maximise the impact that RAMPART participants would make to the evidence base for adjuvant ICI therapy. No information from the research arms was used in any of these design modifications.

The RAMPART TMG requested and received permission from the trial's IDMC to release the Kaplan Meier DFS curve for the control arm, along with numbers at risk. Data from the two research arms were not requested or made available. The control arm information was used by the RAMPART statisticians, alongside the observed accrual pattern, to check the control arm event rate assumptions and sample size estimates made at the start of the trial, and to plan the estimated timelines for the revised targeted effect size.

To consider durvalumab or the combination of durvalumab with tremelimumab as alternative options to pembrolizumab, the observed treatment effect would have to be as good as, or better than that observed in KEYNOTE-564 (HR for DFS of 0.63 (95 % CI 0.50–0.80)). In the revised RAMPART design a HR_BvsA_ = 0.60 is targeted for durvalumab monotherapy versus control (arm B vs. arm A) and a HR_CvsA_ = 0.55 is targeted for the combination arm versus control (arm C vs. arm A). The HR of 0.60 for durvalumab monotherapy is a slightly larger target effect size than was observed in KEYNOTE-564 for pembrolizumab monotherapy; the HR of 0.55 for durvalumab plus tremelimumab reflects that a larger effect size would be expected when participants are treated with an additional agent.

In the modified design, the originally planned interim analyses were removed; with recruitment closing earlier than planned, the required number of control arm events for these analyses would not be met.

As the primary outcome of DFS is a time-to-event measure, the timing of each analysis is driven by the required number of control arm events. Based on the existing control arm event data that had been observed up to November 2022, we predicted the first planned analysis of arm C vs arm A to be timed for June 2026. As this was thought to be quite some time in the future, the TMG requested the addition of a superiority analysis, assessing the combined research arms (B and C) against the control (A). This would permit an early read-out on the potential efficacy of treating patients with either durvalumab or durvalumab with tremelimumab while we waited on the results from the individual arms comparisons; it would also help to guide the follow-up plan for all randomised participants. The target HR for this analysis was set to HR_B + CvsA_ = 0.575 (the average of the target HRs of B vs. A and C vs. A respectively).

We set the revised overall power to 80 % and maintained the family-wise error rate (FWER) at 2.5 % (one-sided) as per the original design. A closed testing procedure at the time of the combined superiority analysis (arms B + C vs. arm A), would allow us to interpret the results of arm C vs. arm A and arm B vs. arm A, respectively, in light of having conducted the combined analysis earlier. This naturally preserves the FWER. To obtain the critical value for the two pairwise comparisons and control the FWER at 2.5 %, we used Dunnett's method. This enables us to account for the correlation between the two test statistics as both analyses are using the same control arm patients. The resulting, Dunnet-corrected, one-sided alpha equals to 1.29 %.

Table 1 presents the estimated timepoint of each analysis in the modified design, with the associated significance level, power and required number of control arm events. The primary analysis of arms B + C vs. arm A was planned when we would observe 59 control arm events, which was expected to occur five and a half years from the start of recruitment. The analysis of arm C vs. arm A would follow when 91 control arm events were observed, 8 years after the start of recruitment (June 2026). If there was a significant result (i.e., the resulting p-value is less than 1.29 %), we would have committed a pairwise and a family-wise error under the null hypothesis. Based on the definition of FWER which states that at least one pairwise error occurs during the trial, we could revert to the same significance level as the combined superiority analysis and test arm B vs. arm A at the one-sided 2.5 % level. This would control the FWER for the entire study, and the pairwise error rate for each pairwise comparison at 2.5 % one-sided. The analysis of arm B vs arm A requires 102 control arm events; it was predicted to take place 9.25 years post the start of recruitment (September 2027). On the other hand, if we did not observe a statistically significant result for arm C vs. arm A at 1.29 %, the primary DFS analysis of arm B vs. arm A would occur when we obtained 119 control arm events, which was estimated to happen approximately 11 years after the start of recruitment (June 2029).

**Table 1:** Characteristics and control arm events for the original and modified trials designs.

Parameter		Original Design	First Modification	Final Design
**Recruitment duration**		5.5 years	5 years	5 years
**Sample size**		1750	790	790
**Primary outcome**		DFS OS (high-risk patients only)	DFS	DFS
**Interim analyses**		3 for B vs. A 1 for C vs. A	0	0
**Superiority Analyses**		C vs. A B vs. A	B + C vs. A C vs. A B vs. A	C vs. A B vs. A
**Target hazard ratios (HRs)**		C vs. A: 0.7 B vs. A: 0.75	B + C vs. A: 0.575 C vs. A: 0.55 B vs. A: 0.60	C vs. A: 0.55 B vs. A: 0.60
**Target control arm events**	B + C vs. A	Not Applicable	59	Not Applicable
	C vs. A	276	91	91
	B vs. A	416	102 (if C vs. A not significant) 119 (if C vs. A significant)	102 (if C vs. A not significant) 119 (if C vs. A significant)
**Alpha and Power for each Comparison**	B + C vs. A	Not Applicable	0.025, 0.80	Not Applicable
	C vs. A	0.0097, 0.87	0.0129, 0.82	0.0129, 0.82
	B vs. A	0.0097, 0.835	0.025, 0.82 (if C vs. A not significant) 0.0129, 0.80 (if C vs. A significant)	0.025, 0.82 (if C vs. A not significant) 0.0129, 0.80 (if C vs. A significant)
**FWER, one-sided**		0.025	0.025	0.025
**Overall power**		0.90	0.80	0.80

## 7 RAMPART revised analysis timeline – update in December 2023

The predicted time of analysis for arms B + C vs arm A was December 2023. Follow-up data continued to be collected from all participating sites and the target number of control arm events was being monitored in preparation for analysis. In December 2023, the target number of control arm events (59) was reached. However, despite best efforts, compliance with data completion was less than adequate. In January 2024, the TMG agreed to postpone the analysis of Arms B + C vs Arm A until later in 2024 to allow for a more intensive chase of follow-up data. This decision was made prior to any analysis, and without knowledge of any data from the research arms.

## 8 RAMPART revised analysis timeline – update in July 2024

In July 2024, the trial statistician reviewed the number of control arm events in preparation for the analysis of arms B + C vs arm A. A huge effort from the trial team and site staff to improve data return rates meant that 81 control arm events had been reported, including many which had occurred in earlier years of the trial. This meant that we were much closer to the number needed for the primary analysis of the pairwise comparison of arm C vs arm A (91). Based on the most up to date control arm survival rates, the analysis timepoint was re-estimated to occur much earlier than the original prediction (December 2024, compared to June 2026). This development removes the need for the analysis of arms B + C vs arm A, which had only been added to provide an early readout on the potential efficacy of the research interventions. Without access to any data from the research arms, the TMG unanimously agreed to delay the primary analysis until the target number of control arms for the arm C vs arm A analysis is reached.

Removing the combined arm B + C v arm A analysis has no effect on the power and significance levels of the pairwise comparison analyses (arm C vs arm A and arm B vs arm A respectively). Since the Dunnett's multiplicity adjustment had been used to control the overall Type I error rate (FWER) across the two pairwise comparisons after the combined analysis of arms B + C vs arm A, the FWER is still controlled at α_FWER_ = 2.5 % (one-sided) with the overall pairwise power of 80 %. The Dunnett-corrected significance level for the pairwise comparisons remains the same, at α_Dun_ = 1.29 % one-sided. Given the definition of FWER i.e. the probability of committing at least one type I error (incorrectly rejecting the null hypothesis for at least one pairwise comparison), and the statistical significance of the arm C vs arm A analysis, we will be able to analyse arm B vs arm A using either α_FWER_ (arm C vs arm A statistically significant, analysis timepoint June 2025) or α_Dun_ (arm C vs arm A not statistically significant, analysis timepoint February 2026) (see Table 1). The predicted timelines are based on the most recent data and are subject to change as the accumulating data matures. The trial team will monitor this and update the analysis timelines accordingly.

## 9 The continued relevance of RAMPART

When we initiated RAMPART, the standard of care for RCC patients, post nephrectomy, was active monitoring. The landscape has now changed and pembrolizumab is approved and in use based on the KEYNOTE-564 trial results. One substantial limitation is that KEYNOTE-564, as well as all other adjuvant RCC trials investigating ICI therapy, recruited participants with clear cell (cc) RCC only, thus evidence only exists for clear cell disease.

RAMPART will inform this limitation as participants with both clear and non-clear histological subtypes are eligible for randomisation. RAMPART also includes participants who are at slightly lower risk of recurrence than those included in the other RCC trials. RAMPART will be the only trial to provide any information on the effect of immune checkpoint inhibitors, both as monotherapy and in combination, in these participants. Pre-specified exploratory analyses will therefore be performed to check for any potential for benefit (i.e., in participants with clear and non-clear cell RCC and in intermediate and higher risk participants). A parallel translational research study (TransRAMPART) using tumour and blood samples collected at baseline (after surgery but before commencing RAMPART treatment) and longitudinally will help better predict those patients who will benefit from these treatments. Combining clinical and molecular findings will increase the impact and interest in the RAMPART clinical results.

## 10 Conclusion

Confirmatory clinical trials in the adjuvant setting usually take many years to complete. There is a risk for both academic and pharma-led trials, that other similar trials will report earlier, change treatment options, and affect the ability to complete a trial as planned. Organisations are then faced with trying to ensure the experience of the participants they have randomised will contribute to the evidence base. Because of results external to the RAMPART trial, we have changed the targeted effect sizes and the analysis plan in a blinded and statistically valid way (i.e., without jeopardising the statistical validity of the design) to maximise the contribution that RAMPART participants can make to the evidence base for ICI therapy in the adjuvant RCC setting. In trials with time-to-event outcomes, predictions about the timing of analysis rely on up-to-date information about participants while periodic assessment of the predictions are necessary to provide accurate analysis timelines.

## CRediT authorship contribution statement

**Angela Meade:** Conceptualization, Data curation, Formal analysis, Funding acquisition, Methodology, Project administration, Writing – original draft, Writing – review & editing. **Elena Frangou:** Formal analysis, Methodology, Writing – original draft, Writing – review & editing. **Babak Choodari-Oskooei:** Conceptualization, Formal analysis, Methodology, Writing – original draft, Writing – review & editing. **James Larkin:** Conceptualization, Funding acquisition, Investigation, Writing – original draft, Writing – review & editing. **Tom Powles:** Conceptualization, Funding acquisition, Investigation, Methodology, Writing – original draft, Writing – review & editing. **Grant D. Stewart:** Conceptualization, Investigation, Writing – original draft, Writing – review & editing. **Laurence Albiges:** Conceptualization, Investigation, Writing – original draft, Writing – review & editing. **Axel Bex:** Investigation, Writing – original draft, Writing – review & editing. **Toni K. Choueiri:** Conceptualization, Investigation, Writing – original draft, Writing – review & editing. **Ian D. Davis:** Conceptualization, Writing – original draft, Writing – review & editing. **Tim Eisen:** Investigation, Writing – original draft, Writing – review & editing. **Alison Fielding:** Writing – original draft, Writing – review & editing. **Craig Gedye:** Investigation, Writing – original draft, Writing – review & editing. **David J. Harrison:** Writing – original draft, Writing – review & editing. **Rick Kaplan:** Conceptualization, Methodology, Writing – original draft, Writing – review & editing. **Salena Mulhere:** Writing – original draft, Writing – review & editing. **Paul Nathan:** Writing – original draft, Writing – review & editing, Investigation. **Grisma Patel:** Writing – original draft, Writing – review & editing. **Jay Patel:** Project administration, Writing – original draft, Writing – review & editing. **Hannah Plant:** Project administration, Writing – original draft, Writing – review & editing. **Alastair Ritchie:** Conceptualization, Writing – original draft. **Hannah Rush:** Writing – original draft, Writing – review & editing. **Clare Shakeshaft:** Project administration, Writing – original draft, Writing – review & editing. **Martin R. Stockler:** Conceptualization, Investigation, Writing – original draft, Writing – review & editing. **Cristina Suarez:** Investigation, Writing – original draft, Writing – review & editing. **Jemima Thompson:** Project administration, Writing – original draft, Writing – review & editing. **Nat Thorogood:** Project administration, Writing – original draft, Writing – review & editing. **Balaji Venugopal:** Investigation, Writing – original draft, Writing – review & editing. **Mahesh K.B. Parmar:** Conceptualization, Funding acquisition, Methodology, Writing – original draft, Writing – review & editing.

## Trial registration

ISRCTN #: ISRCTN53348826.

NCT #: NCT03288532.

EUDRACT #: 2017-002329-39.

CTA #: 20363/0380/001-0001.

MREC #: 17/LO/1875.

ClinicalTrials.gov Identifier: NCT03288532.

RAMPART Protocol current version 7.0.

## Funding

Kidney Cancer UK: Non-commercial partner funding

10.13039/100004325AstraZeneca LP: educational grant plus free-of-charge durvalumab and tremelimumab.

Cancer Research UK: Prospective Sample Collection Award

## Declaration of competing interest

The authors declare that they have no known competing financial interests or personal relationships that could have appeared to influence the work reported in this paper.

## Data Availability

The data that has been used is confidential.
